# Palmitoleate Protects against Zika Virus-Induced Placental Trophoblast Apoptosis

**DOI:** 10.3390/biomedicines9060643

**Published:** 2021-06-04

**Authors:** Philma Glora Muthuraj, Aryamav Pattnaik, Prakash K. Sahoo, Md Torikul Islam, Asit K. Pattnaik, Siddappa N. Byrareddy, Corrine Hanson, Ann Anderson Berry, Stephen D. Kachman, Sathish Kumar Natarajan

**Affiliations:** 1 Department of Nutrition and Health Sciences, University of Nebraska-Lincoln, Lincoln, NE 68583, USA; philma@huskers.unl.edu (P.G.M.); psahoo@huskers.unl.edu (P.K.S.); Torikul.nfs.du@gmail.com (M.T.I.); 2 Nebraska Center for Virology, University of Nebraska-Lincoln, Lincoln, NE 68583, USA; aryamavpattnaik@ymail.com (A.P.); apattnaik2@unl.edu (A.K.P.); 3 School of Veterinary Medicine and Biomedical Sciences, University of Nebraska-Lincoln, Lincoln, NE 68583, USA; 4 Department of Pharmacology and Experimental Neuroscience, University of Nebraska Medical Center, Omaha, NE 68198, USA; sid.byrareddy@unmc.edu; 5 Child Health Research Institute, University of Nebraska Medical Center, Omaha, NE 68198, USA; ckhanson@unmc.edu (C.H.); alanders@unmc.edu (A.A.B.); 6 College of Allied Health Professions, University of Nebraska Medical Center, Omaha, NE 68198, USA; 7 Department of Pediatrics, University of Nebraska Medical Center, Omaha, NE 68198, USA; 8 Department of Statistics, University of Nebraska-Lincoln, Lincoln, NE 68583, USA; steve.kachman@unl.edu

**Keywords:** monounsaturated fatty acids, endoplasmic reticulum stress, apoptosis, viral replication, Zika virus, placenta, trophoblast

## Abstract

Zika virus (ZIKV) infection in pregnancy is associated with the development of microcephaly, intrauterine growth restriction, and ocular damage in the fetus. ZIKV infection of the placenta plays a crucial role in the vertical transmission from the maternal circulation to the fetus. Our previous study suggested that ZIKV induces endoplasmic reticulum (ER) stress and apoptosis of placental trophoblasts. Here, we showed that palmitoleate, an omega-7 monounsaturated fatty acid, prevents ZIKV-induced ER stress and apoptosis in placental trophoblasts. Human trophoblast cell lines (JEG-3 and JAR) and normal immortalized trophoblasts (HTR-8) were used. We observed that ZIKV infection of the trophoblasts resulted in apoptosis and treatment of palmitoleate to ZIKV-infected cells significantly prevented apoptosis. However, palmitate (saturated fatty acid) did not offer protection from ZIKV-induced ER stress and apoptosis. We also observed that the Zika viral RNA copies were decreased, and the cell viability improved in ZIKV-infected cells treated with palmitoleate as compared to the infected cells without palmitoleate treatment. Further, palmitoleate was shown to protect against ZIKV-induced upregulation of ER stress markers, C/EBP homologous protein and X-box binding protein-1 splicing in placental trophoblasts. In conclusion, our studies suggest that palmitoleate protects placental trophoblasts against ZIKV-induced ER stress and apoptosis.

## 1. Introduction

Zika virus (ZIKV) was originally identified in the Zika forest of Uganda from the blood sample of febrile macaque [1]. ZIKV infection of humans was first reported to occur sporadically in the African continent in the early 1960s, but later, ZIKV infection spread throughout the world, including Yap islands, Pacific islands, and the American continent between 2007 and 2016 [2]. ZIKV is an arbovirus belonging to the *Flaviviridae* family and is closely related to dengue, chikungunya, and yellow fever viruses [3]. ZIKV usually causes self-limiting disease in a healthy individual, but infection during pregnancy often causes devastating effects to the fetus. ZIKV-infected fetus exhibits congenital Zika syndrome, which is an array of disease manifestations that includes microcephaly, retinal defects, and muscular-skeletal defects [4]. Further, ZIKV infection is also associated with the development of Guillain-Barre syndrome in adults [5].

The placental route plays a major role in ZIKV transmission from the infected mother to the fetus. Studies suggest that placental trophoblasts, endothelial cells, and Hofbauer cells can be infected and play a crucial role in ZIKV dissemination from mother to fetus [6,7,8,9]. ZIKV is also known to cause placental dysfunction by driving an inflammatory condition accompanied by apoptosis [8,10,11]. Further, ZIKV infection of the placental cells was shown to compromise the normal physiological role of the placenta, which is crucial for the fetal growth and survival [12,13,14]. ZIKV can also cause drastic changes in the placental lipid metabolism affecting the nourishment of growing fetus [15].

There are several candidate ZIKV vaccines, which include DNA vaccines, mRNA vaccine, and whole live-attenuated virus or inactivated virus vaccines [16,17,18,19]. Although several candidate vaccines progressed to early clinical trials, they are posed with serious challenges, such as case detection after vaccination and vaccination strategies primarily focusing on females who are already pregnant or expecting pregnancy. Similarly, several drug candidates, such as niclosamide (anti-parasite drug) and emricasan (pan-caspase inhibitor) to prevent ZIKV infection have been identified [20]. However, the safety and administration of niclosamide during pregnancy is under debate (a pregnancy category B drug) and emricasan efficacy is solely attributed to its neuroprotective property rather than its anti-viral activity and has not been tested in pregnant women [21]. Similarly, sofosbuvir, a FDA-approved nucleotide analog and an inhibitor of NS5B polymerase of Hepatitis C virus, was shown to be effective against ZIKV-infected 3 day old Swiss mice model, but still this a pregnancy category B drug, which does not have well-documented studies in humans [22]. With these limitations in hand, in the present study, we tested the novel protective role of palmitoleate against ZIKV-induced trophoblast apoptosis. Palmitoleate (16:1 n-7) is an omega-7 monounsaturated fatty acid and has been shown to protect against saturated free fatty acid-induced apoptosis of hepatocytes, pancreatic beta cells, and umbilical vein endothelial cells [23,24,25]. This led to our hypothesis that palmitoleate prevents ZIKV-induced ER stress and apoptosis in trophoblasts. Our results reveal that palmitoleate protects trophoblasts from ZIKV-induced ER stress and apoptosis by downregulating the levels of ER stress markers, such as CEBP homologous protein (CHOP) and spliced X-box binding protein (XBP1).

## 2. Materials and Methods

### 2.1. Materials

Palmitoleate (#P9417), palmitate (#P5585), fatty acid free BSA (A3803), Steriflip vacuum (# SCGP00525), 4′,6-diamidine-2-phenylindole dihydrochloride (DAPI) (# D9542), and anti-flavivirus group antigen antibody (D1-4G2-4-15 clone) (# MAB 10216) were purchased from Millipore Sigma, Burlington, MO, USA. Apo-ONE homogenous caspase 3/7 assay (WI # G7791) was obtained from Promega, Madison, WI, USA. Alexaflour-488 conjugated anti-mouse antibody (#A11001), TRIzol Reagent (# 15596018), Superscript II reverse transcriptase, (#18064-014), RNaseOUT (#10777-019), Random Hexamers (#8080127) were obtained from Thermo Scientific, Waltham, MA, USA and QIAamp viral-RNA isolation kit was from Qiagen, Hilden, Germany (#52906). Hydrolysis probe for Viral E protein detection were custom synthesized by IDT, IA. Restriction enzyme, Pst I was from New England Biolabs Ipswich, MA, USA (MA #R0140).

### 2.2. Cells

JEG-3 and JAR cells, human choriocarcinoma-derived third trimester trophoblast cell lines, HTR-8SV/neo (HTR-8), a first trimester human immortalized trophoblasts, and Vero cells were used. MEM for JEG-3 cells and DMEM for JAR and HTR-8 cells containing 10% fetal bovine serum and 0.01 % plasmocin was used for. Vero cells were used for plaque assay and cultured in DMEM (Gibco, Waltham, MA, USA) containing, sodium bicarbonate (3.7 g/L), 1X penicillin and streptomycin, 10% FBS, and 0.01% plasmocin. All cells used in the present study were obtained from ATCC and periodically tested for mycoplasma.

### 2.3. Virus and Infection of the Trophoblasts

Original Ugandan MR766 strain (MRV) or recombinant MR strain of Zika virus (r-MRV) [26] or PRVABC59 (PRV, Asian lineage isolated from Puerto Rico) were used to infect the cells with 0.1–1 MOI. Virus infection media containing 2× DMEM (Gibco), 2% fetal bovine serum, 100 µg/mL streptomycin, 100 I.U./mL penicillin, 7.5% sodium bicarbonate, 20 mM HEPES, sodium pyruvate, 1× nonessential amino acids, and 0.01% plasmocin was used. After virus adsorption, the media was replaced with DMEM or MEM containing 10% fetal bovine serum and 1% BSA.

### 2.4. Treatment of Fatty Acids

Palmitoleate and palmitate were dissolved in isopropanol with a stock solution concentration of 80 mM. Fatty acid-free BSA (1%) was dissolved in growth media at room temperature using a tube rotator and incubated at 37 °C for 30 min in a water bath and then filter sterilized. Fatty acids were then incubated in the freshly prepared 1% fatty acid-free BSA for fatty acid-BSA conjugation by incubating at 37 °C in the water bath for 20 min. We have used 100–200 µM concentrations of fatty acids for 48–96 hpi.

### 2.5. Biochemical and Structural Characterization of Apoptosis

Structural and biochemical markers of apoptosis like percent apoptotic nuclei and caspase 3/7 activity, respectively, were assessed. Percent apoptotic nuclei was quantified by characteristic nuclear morphology and visualized by treatment with the fluorescent DNA-binding dye, DAPI as described [27]. Briefly, cells were stained with 5 μg/mL of DAPI for 5–10 min at 37 °C. Apoptotic nuclei (condensed, fragmented) were counted and presented as a percent of total nuclei. At least 100 cells were counted per well and experiments were performed in triplicate. Caspase 3/7 activity was measured using rhodamine 110 bis-(N-CBZ-L-aspartyl-L-glutamyl-LI-valyl-aspartic acid amide (Z-DEVD-R110) substrate. The caspase 3 and 7 enzyme activity in the cells will cleave the DEVD peptide in the substrate and release the rhodamine 110 fluorophore, which can be measured spectrofluorometrically (BioTek Synergy, Winooski, VT, USA) with a 498 nm wavelength of excitation and 521 nm emission. The data were reported as fold-change of net fluorescence compared to vehicle treated cells, with experiments performed in triplicate or quadruplicate.

### 2.6. Immunofluorescence Analysis

After 48 h of ZIKV infection, the media was aspirated and the cells were washed with phosphate buffered saline (PBS). Fixing was done with methanol and acetone at the ratio of 1:1 and washed thrice with PBS. Primary antibody, anti-flavivirus group antigen antibody (D1-4G2-4-15 clone) was used at a dilution of 1:500 and incubated at room temperature for 2 h with gentle rocking. After primary antibody incubation, cells were washed thrice with PBS in 5 min intervals. Alexaflour-488 conjugated anti-mouse antibody (Invitrogen, Carlsbad, CA, USA) was added at a dilution of 1:1000 and kept in a shaker at room temperature for 1 h. After incubation, the cells were washed thrice in PBS and then visualized under Nikon A1R-Ti2 confocal system.

### 2.7. Quantitative Real Time Polymerase Chain Reaction

Total RNA was extracted from cells, 48–96 hpi using TRIzol reagent as described in the manufacturer protocol. Around 1–5 µg RNA from each sample was reverse transcribed to cDNA using random hexamers, RNaseOUT, and Superscript II. Relative CHOP mRNA expression was quantified using Light cycler 480 SYBR Green I Master Version 13 (Roche, Basel, Switzerland) in a Bio-Rad CFX connect Real-Time System (Hercules, CA, USA). CHOP published primers as described [28] were used. The housekeeping gene 18S rRNA was used as a control, and the primer used, are listed in Table 1.

### 2.8. Quantification of Viral RNA Copy Number Using Hydrolysis Probe

RNA form cell culture supernatant was isolated by QIAamp viral-RNA extraction kit. RNA from cell lysate was isolated by TRIzol and cDNA synthesis was performed. TaqMan hydrolysis probes for quantitative PCR primers targeting E gene of the ZIKV was used (Table 1). Absolute RNA quantification was performed using a standard curve generated from the PCR product using primers listed in Table 1, and as described in [26].

### 2.9. XBP1 mRNA Splicing Assay

The cDNA samples with 1:3 or 1:10 dilution was subjected to PCR to amplify *XBP1* gene using the primer set (each 20 µM), as described [29]. The PCR product (around 8 µL) was digested with 1 µL of PstI (20 U) in 1 µL of 3.1 NEB buffer containing 100 mM NaCl, 50 mM Tris-HCl, 10 mM MgCl_2,_ 100 µg/mL BSA pH 7.9, and incubated at 37 °C for 2 h. The restriction enzyme digested PCR product was electrophoresed in 2% agarose gel stained with ethidium bromide. The unspliced 474 bp nucleotide and can be cleaved by PstI enzyme by recognition of the intact restriction site, resulting in 296 bp and 183 bp fragments. The spliced forms lack the intact restriction enzyme site so the bands are visualized around 448 bp. GAPDH was used as a control and was amplified using primers listed in Table 1 [30]. Relative band intensities were analyzed using Image J software.

### 2.10. Western Blot

Cell lysates were scrapped using 100 µL of lysis buffer made of 50 mM Tris pH 7.4, 150 mM NaCl, 1 mM EDTA, 1 mM DTT, 1 mM Na_3_Vo_4_, 1 mM PMSF, 100 mM NaF, and 1% Triton x-100. Cell supernatant obtained after 10,000× *g* for 10 min of centrifugation and was used for protein estimation using the Pierce Modified Lowry 660 nm protein assay reagent (Thermo Fisher Scientific, Waltham, MA, USA). Around 30 µg protein was electrophoresed in a 10% SDS-polyacrylamide gel and transferred into nitrocellulose membrane. The membrane was blocked with 5% BSA in TBST. Primary antibody was used in 1:1000 dilution, with 5% BSA in TBST. Secondary antibody was used in 1:5000 dilution. Washes of 10 min each for 3 times were employed after both primary and secondary antibody incubation. The blot was developed using Clarity Western ECL substrate (Bio-Rad, Hercules, CA, USA).

### 2.11. Plaque Assay

The plaque assay was performed as described [31]. Briefly, cell culture supernatants were collected after 48 h of ZIKV infection and fatty acid treatment (48 hpi) and diluted serially 10^−3^ to 10^−4^ using virus infection media in duplicates. The diluted supernatant samples were kept for virus adsorption for 1 h over 90% confluent Vero cells and the cells were washed with PBS prior to virus adsorption. After virus adsorption, 1:1 ratio of 2% low melting agarose and plaque assay media containing 2× DMEM, 1× penicillin and streptomycin, 4% FBS, sodium bicarbonate 7.5%, 20 mM HEPES, sodium pyruvate, 1X nonessential amino acids, and 0.01% plasmocin were added to each well and incubated at 37 °C for 4 days. Fixation of cells was done by using 10% formalin in PBS for 1 h. After the removal of agarose overlaid in each well, fixed cells were stained with 0.1% crystal violet staining solution for 1 h, followed by washing the plates with distilled water and allowing it to air dry. Plaques were counted in each well and expressed plaque forming units/mL (pfu/mL).

### 2.12. Percent Cell Survival Using 3-(4,5-dimethylthiazol-2-yl)-2,5-diphenyltetrazolium Bromide (MTT)

Stock solution containing 50 mg of MTT in 10 mL of PBS was filter sterilized and stored at 4 °C. Approximately 20 µL of MTT stock was diluted to 100 µL in MEM media without any FBS. Media from the cells grown in a 96-well plate was aspirated and then 110 µL of the prepared MTT solution was added to each well. After 4 h of incubation at 37 °C, media was aspirated, and 100 µL of isopropanol containing 1 µL 37% HCl per 10 mL of isopropanol was added to each well. The contents were gently mixed, and the absorbance was measured at 540 nm.

### 2.13. Assessment of Cell Viability Using Crystal Violet

The assay was performed as described by [32]. Briefly, in a 24-well plate 30,000 cells were seeded and infected with ZIKV, and fatty acids were treated after 1 h of virus adsorption. After 48 h, media was aspirated and washed two times with double distilled water. The cells were then stained with 300 µL of 0.5% crystal violet staining solution per well, for 20 min at room temperature, with gentle rocking. The excess stain after incubation was washed four times with double distilled water and air-dried for 2 h by inverting the plate on to a blotting paper. Once the plate was dried, around 500 µL of methanol was added to each well and placed on a rocker for 20 min at room temperature, and the absorbance was measured at 570 nm.

### 2.14. Data Analysis

Data are expressed as mean ± standard error of mean (SEM). Statistical analysis was performed using Welch’s *t*-test, *p* value < 0.05 was considered as statistically significant.

## 3. Results

### 3.1. Palmitoleate Prevents ZIKV-Induced Placental Trophoblast Apoptosis

**Figure 1:**
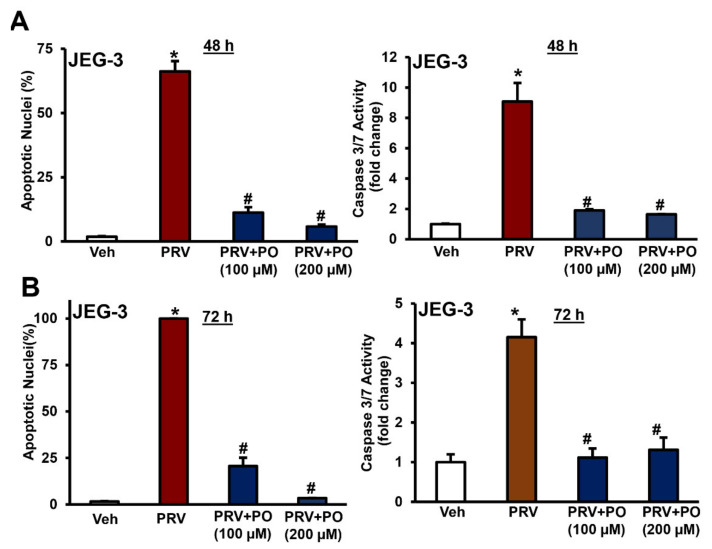
Palmitoleate treatment after Zika virus (ZIKV) PRVABC59 strain (PRV) infection inhibits apoptosis in JEG-3 cells. (**A**) Structural and biochemical characterization of apoptosis in JEG-3 infected with the 1.0 MOI (multiplicity of infection) ZIKV PRV strain, 48–72 h showed an increase in percent apoptotic nuclei compared to uninfected vehicle cells. Infected cells treated with 100 or 200 µM of palmitoleate, 48–72 hpi (hours post infection) showed a decrease in the percent apoptotic nuclei when compared to ZIKV-infected cells alone (left panels). (**B**) There was a significant activation of caspase 3/7 in ZIKV-infected cells, but caspase 3/7 activation was significantly blocked when the infected cells were treated with 100 or 200 µM of palmitoleate both at 48 and 72 hpi (right panels). Data represent mean ± SEM, *n* = 3. * *p* < 0.05 compared to uninfected vehicles cells, # *p* < 0.05 compared to ZIKV-infected cells.

We observed the characteristics apoptotic nuclear morphological changes in ZIKV-infected placental trophoblasts and showed a dramatic increase in percent apoptotic nuclei after 1.0 MOI of PRV infection for 48–96 h in JEG-3 cells (Figure 1A,B and Supplementary Figure S1A). Percent apoptotic nuclei significantly reduced with the treatment of 100–200 µM of palmitoleate after 1 MOI of PRV infection for 48–96 h in JEG-3 cells compared to ZIKV infection alone (Figure 1A,B, Supplementary Figure S1A). Similarly, treatment of ZIKV-infected trophoblasts with palmitoleate significantly downregulated caspase 3/7 activity after 48–72 h of infection compared PRV infection alone in JEG-3 cells (Figure 1A,B).

**Figure 2:**
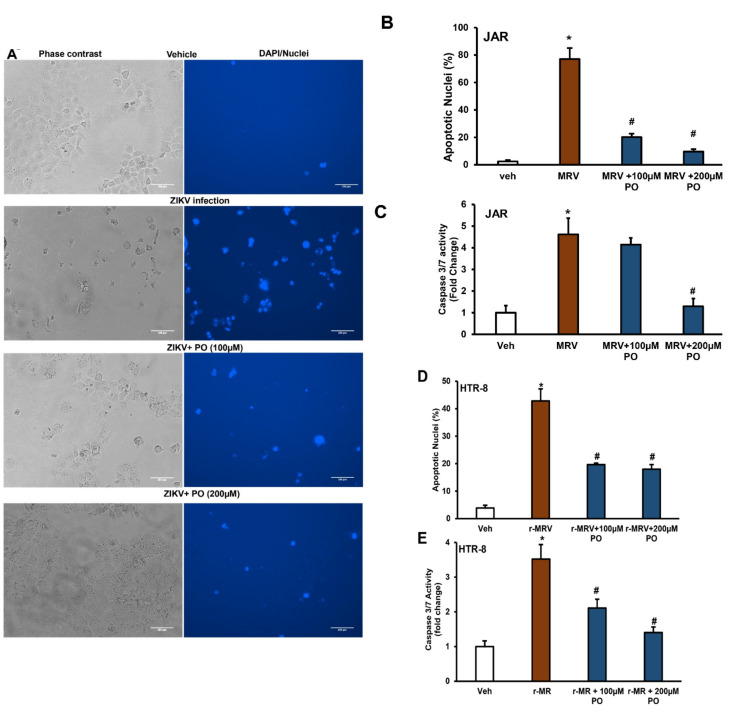
Treatment of the placental cells with palmitoleate after MR766 (MRV) strain of Zika virus (ZIKV) infection inhibits apoptosis. (**A**) Phase contrast (left panels) and DAPI stained (right panels) image panels: characteristic nuclear morphological changes were detected with DAPI staining in third trimester-derived JAR cells after 48 hours post-infection (hpi) with MRV. Vehicle cells show little to no DAPI staining. Infected panel shows higher number of DAPI stained fragmented and condensed nuclei compared to 100 or 200 µM palmitoleate treated cells. The images shown here are representative images. (**B**) JAR cells infected with 1.0 MOI MRV strain of ZIKV showed increase in the percentage apoptotic nuclei when compared to uninfected vehicle cells. In cells treated with 100 and 200 µM palmitoleate 48 hpi showed a significant decrease in the percent apoptotic nuclei compared to ZIKV-infected cells. (**C**) There was a significant increase in caspase 3/7 activity in MRV-infected cells, but this was significantly blocked when the cells were treated with 200 µM of palmitoleate post-infection and a trend towards downregulation at 100 µM palmitoleate treatment in the MRV-infected cells. (**D**) Characteristic nuclear morphological changes were detected using DAPI staining in the first trimester derived HTR-8 cells after 72 hpi were calculated and represented as percent apoptotic nuclei. Cells infected with 1.0 MOI r-MRV (recombinant MR766) strain of ZIKV showed an increase in the percent apoptotic nuclei when compared to uninfected vehicle cells. However, cells treated with 100 and 200 µM of palmitoleate post-infection showed a significant decrease in the percentage apoptotic nuclei compared to ZIKV-infected cells. (E) There was an increase in caspase 3/7 activity in ZIKV infected cells when compared to uninfected vehicle cells, but this activation was significantly blocked when the cells were treated with 100 or 200 µM of palmitoleate, 72 hpi. Data represent mean ± SEM, *n* = 3 for percent apoptotic nuclei, and *n* = 4 for caspase 3/7 activity. * *p* < 0.05 compared to uninfected vehicles cells, # *p* < 0.05 compared to ZIKV-infected cells.

We next tested the protective role of palmitoleate in another human term-derived placental trophoblast (JAR cells) using MRV, the original Ugandan strain for 48 h. JAR cells infected with MRV showed dramatic increase in the number of cells that show fragmented and condensed nuclei compared to vehicle cells. Increased nuclear morphological changes were prevented with the treatment of palmitoleate in trophoblasts (100–200 µM, Figure 2A). Further, increased percent apoptotic nuclei observed in 1.0 MOI MRV infection was dramatically reduced with the treatment of 100 and 200 µM palmitoleate in JAR cells (Figure 2A,B). We also observed a significant decrease in caspase 3/7 activity with 200 µM of palmitoleate treatment and a trend in decreased caspase 3/7 activity with the 100 µM of palmitoleate treatment in 1.0 MOI MRV-infected trophoblasts (Figure 2C). Similarly, JAR cells infected with 1.0 MOI of PRV also showed increased caspase 3/7 activity and this was prevented with the treatment of 100–200 µM of palmitoleate (Supplementary Figure S1B).

To test whether palmitoleate would also protect against first trimester derived placental trophoblast cells, we used HTR-8 cells. Similar to JEG-3 and JAR cells, HTR-8 cells showed enhanced apoptosis as evidenced by an increase in the levels of percent apoptotic nuclei and caspase 3/7 activity with 1.0 MOI of r-MRV for 72 h (Figure 2D). Treatment of 100 and 200 µM palmitoleate to 1.0 MOI of r-MRV-infected HTR-8 cells significantly reduced the percent apoptotic nuclei and caspase 3/7 activation (Figure 2E).

### 3.2. Treatment of Palmitoleate to ZIKV-Infected Trophoblasts Reduces Viral RNA Copy Number

**Figure 3:**
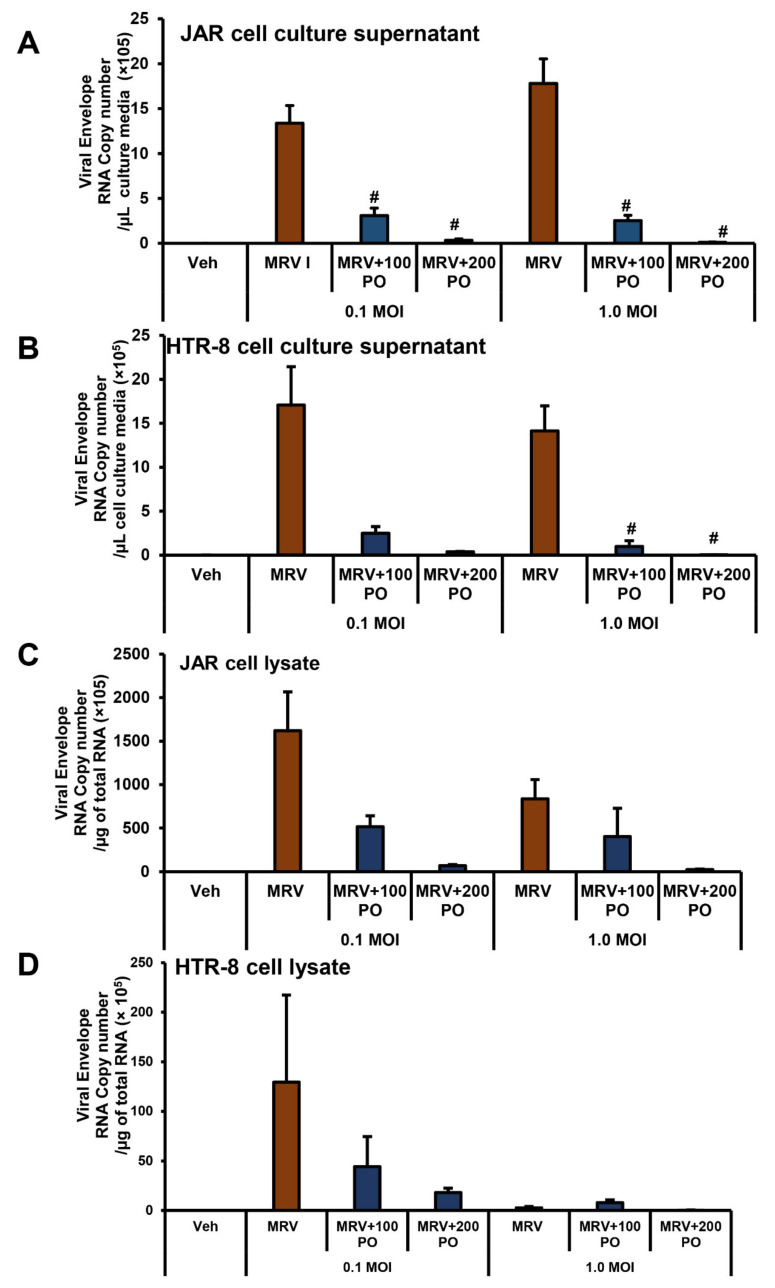

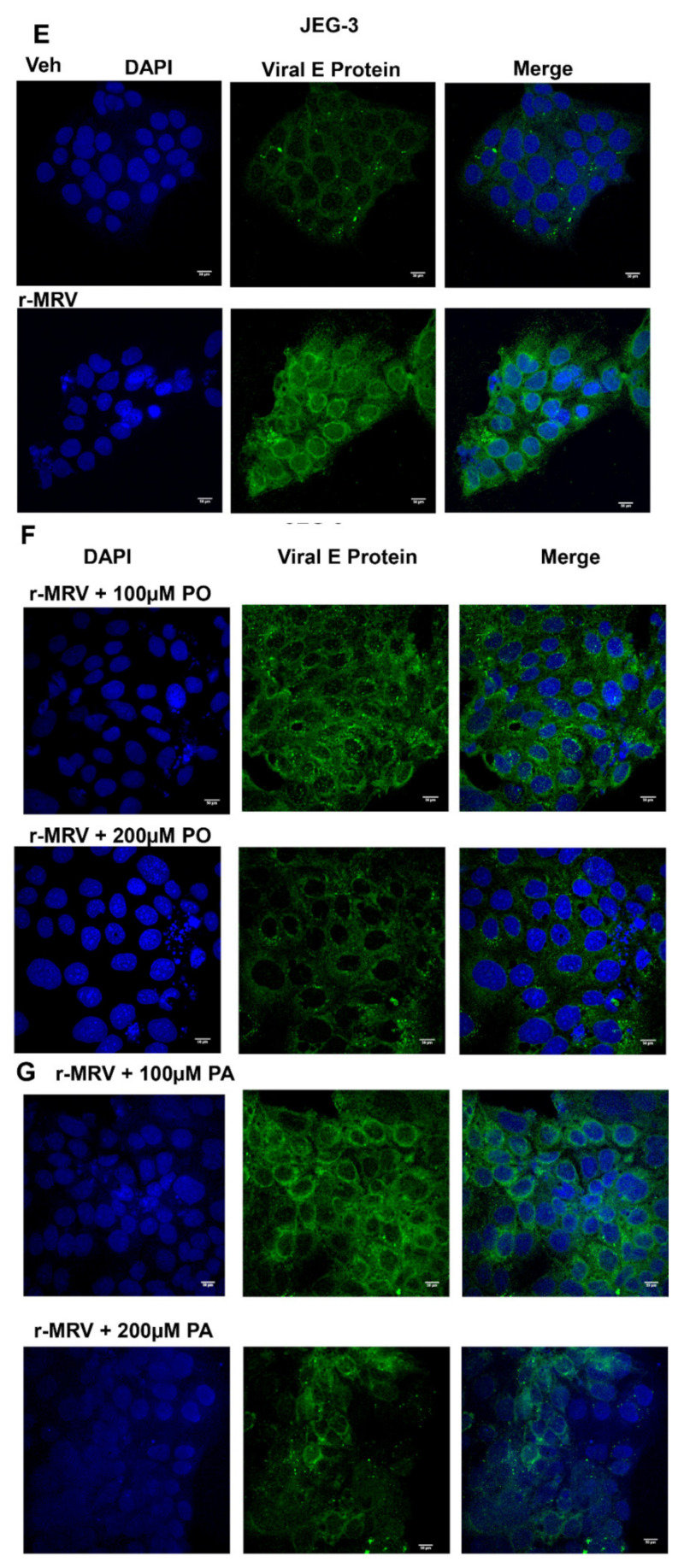

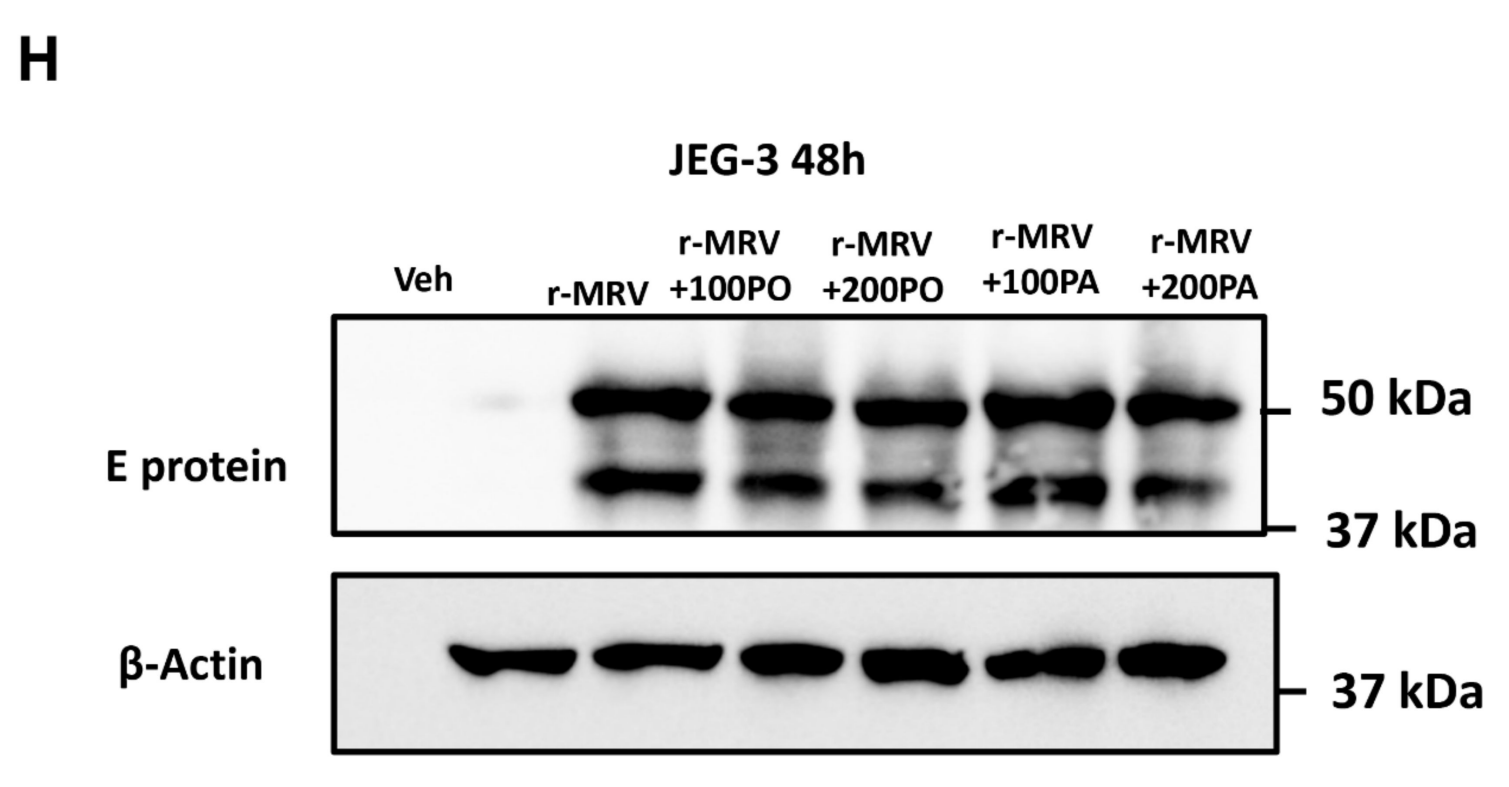
Treatment of the placental trophoblasts with palmitoleate after Zika virus (ZIKV) infection interferes with viral replication. The presence of the viral envelope RNA copy number in the cell culture supernatant of both (**A**) JAR and (**B**) HTR-8 cells infected with 0.1 and 1.0 MOI of ZIKV MR766 strain 72 h and 96 hpi, respectively. When MRV-infected cells were treated with palmitoleate (100–200 µM), there was significant reduction in the viral envelope RNA copy number in the cell culture supernatant with both 0.1 and 1 MOI in JAR cells and with 1 MOI in HTR-8 cells (**A**,**B**). The presence of viral envelope RNA copy number both in JAR (C) and HTR-8 (**D**) cell lysate infected with 0.1 and 1.0 MOI of ZIKV MR766 strain 72 and 96 hpi, respectively. When the infected cells were treated with 100–200 µM of palmitoleate, there was a trend showing reduced viral envelope RNA per microgram of total RNA in both JAR cell lysate (**C**) and HTR-8 cell lysates (**D**). Data represent mean ± SEM, *n* = 3. # *p* < 0.05 compared to ZIKV-infected cells. (**E**–**G**) Viral E protein staining in JEG cells infected with 0.1 MOI r-MRV and in cells treated with 100 or 200µM palmitoleate, 48 hpi. (**E**) ZIKV-infected cells show green fluorescence indicating presence of viral E protein. (**F**) ZIKV-infected JEG-3 cells treated with 200 µM palmitoleate and show a reduction in the intensity of viral E protein staining. (**G**) Infected JEG-3 cells treated with 100–200 µM palmitate showed increased intensity of viral E protein staining. Nuclei were stained with DAPI. (**H**) Immunoblot analysis showed a dramatic increase in viral E protein expression with ZIKV infection in JEG-3 cells compared to vehicle uninfected cells. Treatment of 200 µM palmitoleate to ZIKV-infected cells showed reduced viral E protein expression compared to the ZIKV-infected cells alone.

JAR cells were infected with MRV for 72 h and palmitoleate treatment (100 and 200 µM) showed a dramatic decrease in the Zika viral (E gene) RNA copy number in cell culture supernatant with both 0.1 and 1 MOI, and a trend towards a decrease in cell lysate (Figure 3A,C). This suggests that palmitoleate interferes with ZIKV replication and its release from infected placental trophoblasts. Similarly, HTR-8 cells infected with MRV for 96 h showed a significant reduction in viral envelope RNA copy numbers with the treatment of palmitoleate (100 and 200 µM) with 1 MOI infection and a trend towards reduction with 0.1 MOI in cell culture supernatant (Figure 3B). Palmitoleate treatment with both 0.1 and 1 MOI in HTR-8 cells showed only a trend towards reduction in the viral RNA copy number in the cell lysate (Figure 3D).

#### Immunofluorescence Analysis of Zika Viral E protein

We next tested the expression of viral E protein in JEG-3 cells 48 hpi with 0.1 MOI of r-MRV. There was an increase in the expression of Zika viral E protein in JEG-3 cells with 0.1 MOI r-MRV infection compared to the uninfected vehicle cells (Figure 3E). The viral E protein expression was dramatically reduced in JEG-3 cells treated with palmitoleate (200 µM) 48 hpi compared to ZIKV-infected cells alone. The viral E protein expression dramatically increased in JEG-3 cells treated with palmitoleate (100 µM) 48 hpi and palmitate (100–200 µM) (Figure 3F). The number cells in the 200 µM palmitate treated cells was significantly less, which was comparable to infected cells alone (Figure 3G). Western blot analysis of JEG-3 cells infected with 0.1 MOI r-MRV showed dramatic increase in viral E protein and treatment of 100–200 µM palmitoleate showed a decrease in viral E protein expression. Treatment of palmitate (100–200 µM) did affect the viral E protein expression 48 hpi (Figure 3H). Similarly, there was a non-significant decrease in pfu/mL in cell culture supernatant from 200 µM palmitoleate treated cells, 48 hpi in JEG-3 cells.

Palmitate treatment at 100 or 200 µM concentration altered JEG3 viral load in cell culture supernatant (Supplementary Figure S2A). Palmitoleate treatment (100–200 µM) in JEG-3 cells after infection with 0.1 MOI r-MRV showed a non-significant decrease in the viral (E gene) RNA copy number in the cell lysate. We also observed a non-significant decrease in viral E gene RNA copy number in the cell lysate with 100 µM palmitate treatment. However, JEG-3 cell lysate after 200 µM palmitate treatment to 0.1 MOI r-MRV-infected cells showed a significant reduction in the viral RNA copy number compared to ZIKV-infected cells alone (Supplementary Figure S2B).

### 3.3. Palmitate Does Not Protect Against ZIKV-Induced ER Stress and Apoptosis

**Figure 4:**
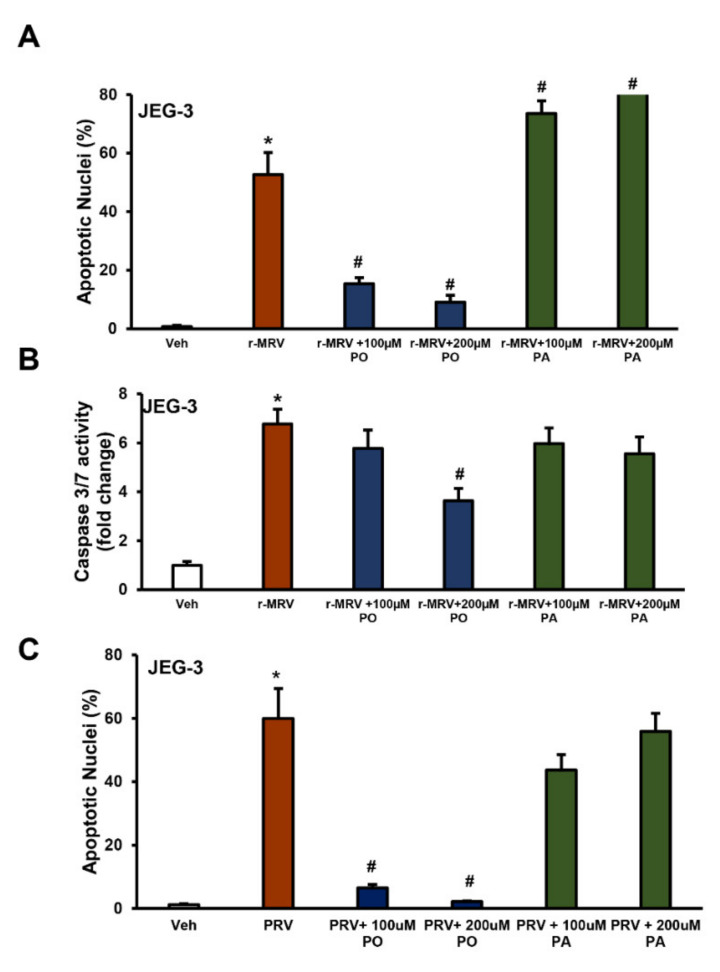
Palmitoleate protects against ZIKV-induced apoptosis but not palmitate. (**A**,**B**) There was significant reduction in (**A**) percentage apoptotic nuclei and (**B**) caspase 3/7 activation in JEG-3 cells treated with palmitoleate 48 hpi with 0.1 r-MRV suggesting the protective role of palmitoleate against ZIKV-induced apoptosis. When the JEG-3 cells were treated with palmitic acid 48 hpi, there was significant increase in the percent apoptotic nuclei and a trend towards increase in caspase 3/7 activation, suggesting that palmitate did not protect against ZIKV-induced apoptosis. Data represent mean ± SEM, *n* = 3 for percentage apoptotic nuclei and *n* = 4 for caspase 3/7 activity. * *p* < 0.05 compared to uninfected vehicles cells, # *p* < 0.05 compared to ZIKV-infected cells. (**C**) Similarly, in JEG-3 cells with 1.0 MOI of PRV for 48 h the percent apoptotic nuclei significantly reduced in 100–200 µM palmitoleate treated PRV-infected cells compared to PRV-infected cells alone; however, treatment of palmitate to ZIKV-infected cells did not prevent ZIKV-induced apoptosis. Data represent mean ± SEM, *n* = 3. * *p* < 0.05 compared to uninfected vehicles cells, # *p* < 0.05 compared to ZIKV-infected cells.

Treatment of JEG-3 cells with palmitate, a saturated fatty acid, after ZIKV infection, with r-MR or PR strains, did not protect against ZIKV-induced trophoblast apoptosis (Figure 4). There was a significant reduction in the percent apoptotic nuclei and caspase 3/7 activation with palmitoleate treatment and this protection was not observed with the treatment of palmitate (Figure 4A–C). Additionally, treatment of palmitate to r-MRV-infected JEG-3 cells caused a significant increase in percent apoptotic nuclei levels compared to r-MRV infection alone (Figure 4A). These results suggest a unique protective property of palmitoleate against ZIKV-induced trophoblast apoptosis that are not observed with the treatment of palmitate.

### 3.4. Palmitoleate Improves Cell Viability in ZIKV-Infected Trophoblasts

**Figure 5:**
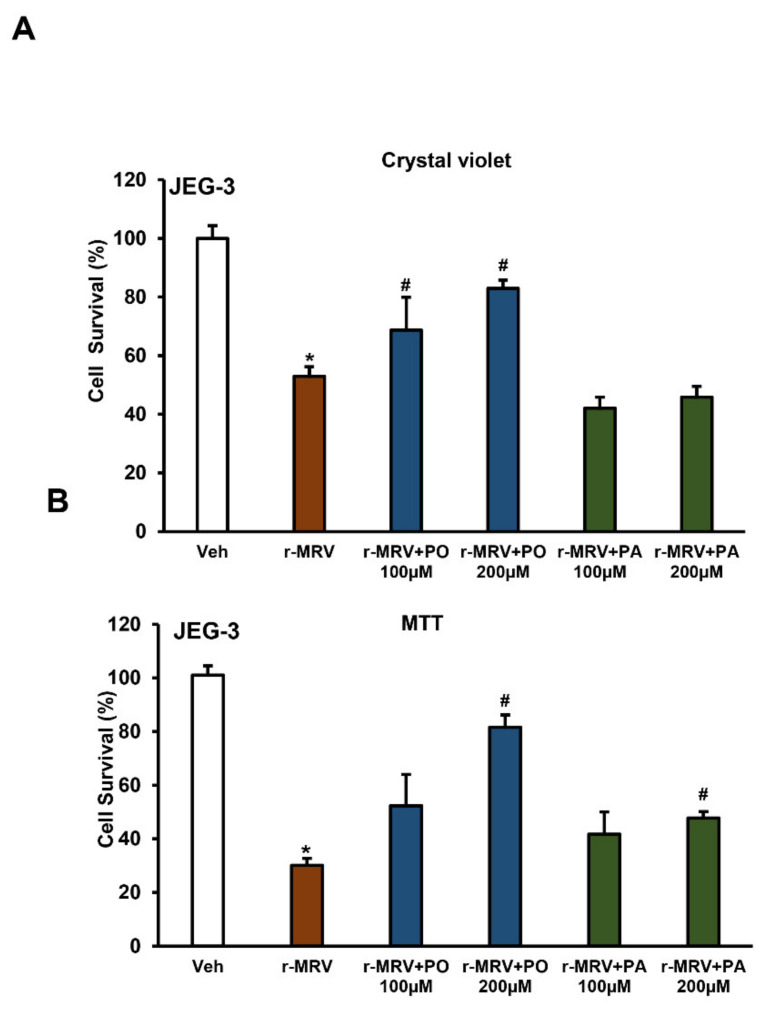
Palmitoleate improves cell viability in Zika virus (ZIKV)-infected cells. (**A**) The percent cell survival using crystal violet assay significantly reduced with 0.1MOI r-MRV when compared to uninfected vehicle cells. The percent cell survival significantly increased with 100 or 200 µM of palmitoleate treatment but was not seen with 100–200 µM palmitate treatment, 48 hpi. (**B**) The percent cell survivability using MTT showed significant improvement in cell survivability with 200 µM palmitoleate treatment in ZIKV-infected cells. Whereas 200 µM palmitate treatment in ZIKV-infected cells also showed a significant increase in cell survivability. Data represent mean ± SEM, *n* = 3 for percent cell viability using crystal violet assay and *n* = 4 for MTT assay. * *p* < 0.05 compared to uninfected vehicles cells, # *p* < 0.05 compared to ZIKV-infected cells.

The cell survival measured using crystal violet shows a significant reduction in the percent cell survival with 0.1 MOI r-MRV infection in JEG-3 cells, 48 hpi when compared to uninfected vehicle cells. The percent cell survival significantly increased with the treatment of 100 or 200 µM palmitoleate post-infection; this protection was not observed with the treatment of palmitate after ZIKV infection (Figure 5A). Similarly, the percent cell survivability assessed using MTT also showed significant reduction in cell survival with 0.1 MOI r-MRV in JEG-3 cells when compared to uninfected vehicle cells. Treatment of palmitoleate to JEG-3 cells infected with 0.1 MOI r-MRV showed a significant increase in cell survivability with 200 µM concentration whereas a 100 µM concentration showed a non-significant increase in percent cell survival. Surprisingly, palmitate treatment in infected cells at 200 µM concentration showed a slight, but significant, increase in percent survival (Figure 5B).

### 3.5. Palmitoleate Protects Against ZIKV-Induced Endoplasmic Reticulum (ER) Stress

**Figure 6:**
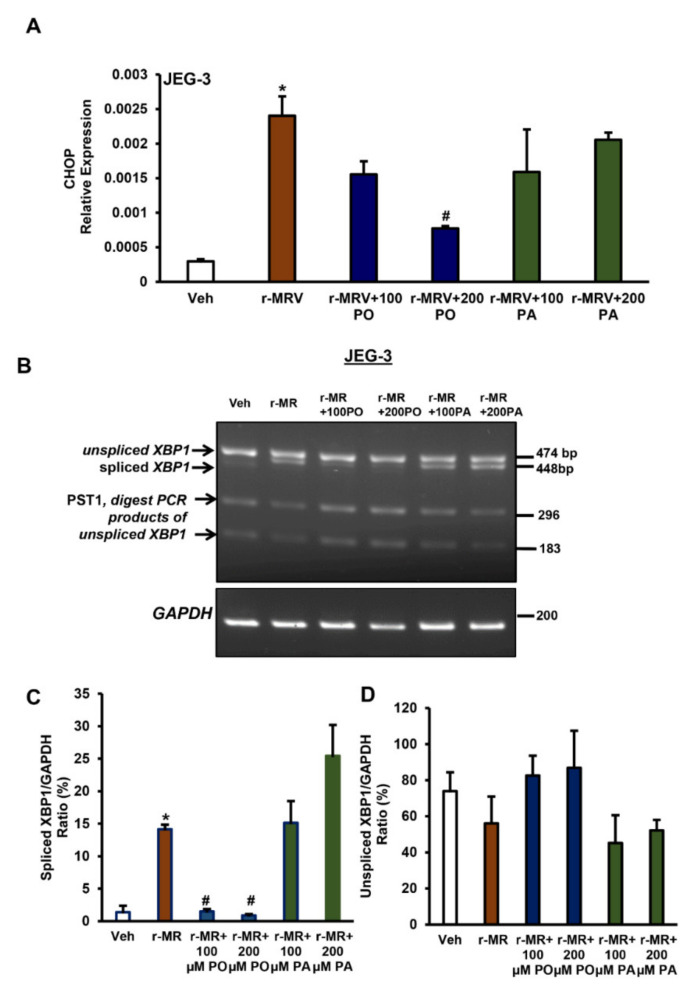
Palmitoleate protects against ZIKV-induced ER stress but not palmitate. (**A**) CHOP mRNA expression was significantly upregulated with 0.1 MOI r-MRV 48 hpi in JEG-3 cells compared to uninfected control cells. There was significant reduction in CHOP mRNA expression in JEG-3 cells treated with 200 µM of palmitoleate 48hpi with ZIKV suggesting protective role of palmitoleate against ZIKV-induced apoptosis and a trend towards decreased expression with 100 µM palmitoleate treatment, but this was not seen with the treatment of 100 or 200 µM palmitate to r-MRV-infected cells. (**B**) Similarly, there was increase in *XBP1* mRNA splicing in infected cells (band ~448 bp) when compared to uninfected vehicle cells. There was significant inhibition of *XBP1* mRNA splicing with the treatment of 100 or 200 µM of palmitoleate in ZIKV-infected cells. However, the protective properties were not seen with the treatment of palmitate to ZIKV-infected cells. (**C**) Quantified levels of spliced XBP1 mRNA (448 bp) relative to GAPDH (Spliced XPB1/GAPDH). (**D**) Quantified levels of unspliced XBP1 mRNA (474 bp) relative to GAPDH (Unspliced XPB1/GAPDH). Data represent mean ± SEM, *n* = 3. * *p* < 0.05 compared to uninfected vehicles cells, # *p* < 0.05 compared to ZIKV-infected cells.

We earlier demonstrated that ZIKV infection of trophoblasts induces an increase in the levels of C/EBP homologous protein (CHOP) mRNA and Spliced X box associated protein-1 (XBP1) mRNA, which are key markers of ER stress [30]. We assessed the activation of ER stress markers, namely CHOP mRNA expression and *XBP1* gene splicing with palmitoleate or palmitate treatment to ZIKV-infected JEG-3 cells. We observed significantly higher levels of CHOP mRNA expression with 0.1 MOI r-MRV infection in JEG-3 cells, 48 hpi compared to uninfected vehicle cells. Treatment of palmitoleate at 200 µM final concentration to the ZIKV-infected cells showed a significant reduction in the expression of CHOP. However, treatment of 100 µM palmitoleate showed only a trend towards a decrease in CHOP mRNA expression compared to ZIKV-infected cells (Figure 6A). Whereas, treatment of 100 or 200 µM palmitate to ZIKV-infected cells did not significantly decrease the expression of CHOP (Figure 6A). We next investigated *XBP1* mRNA splicing levels (~448 bp band), which was an additional indicator of ER stress in cells. Spliced *XBP1* mRNA levels were elevated with 0.1 MOI r-MRV infection in JEG-3 cells compared to uninfected vehicle cells. Palmitoleate treatment (100 or 200 µM) was able to reduce the extensive *XBP1* mRNA splicing seen in the ZIKV-infected cells. However, treatment of palmitate did not prevent the increased levels of spliced XBP1 caused due to ZIKV infection (Figure 6B). The relative band intensity of spliced XBP1 showed a significant increase in percent ratio of spliced XBP1/GAPDH in ZIKV-infected cells when compared to uninfected vehicle cells. There was a trend towards increase in spliced XBP1/GAPDH in palmitate treated cells compared to ZIKV infection alone. However, supplementation of palmitoleate significantly decreased percent ratio of spliced XBP1/GAPDH (Figure 6C). A trend towards increase was also observed with the percent ratio of unspliced XBP1/GAPDH in palmitoleate treated ZIKV infected cells when compared to ZIKV infected cells alone or ZIKV infected cells treated with palmitate (Figure 6D). Thus, treatment of palmitoleate appears to be a protective nutrient therapy against ZIKV-induced ER stress and apoptosis in trophoblasts.

## 4. Discussion

**Figure 7:**
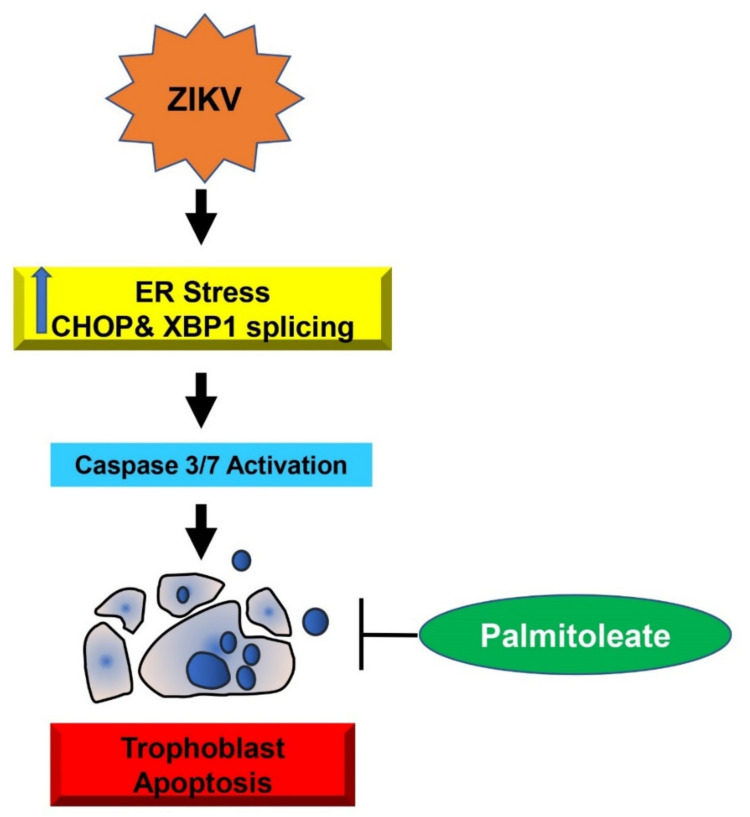
The schematic diagram represents palmitoleate protection against Zika virus (ZIKV)-induced ER stress and apoptosis in placental trophoblasts. ZIKV infection in trophoblasts elicits ER stress via the upregulation of CHOP and XBP1 mRNA splicing, which in turn activates apoptosis. Supplementation of palmitoleate protects against ZIKV-induced ER stress and trophoblast apoptosis.

Zika virus is known to cause apoptosis via sustained ER stress in the trophoblasts [30]. The principal findings of the present study are: (1) palmitoleate, an omega-7 monounsaturated fatty acid significantly reduces ZIKV infection-induced trophoblast apoptosis; (2) treatment of palmitoleate interferes with ZIKV replication in trophoblasts; (3) palmitoleate treatment after ZIKV infection in trophoblasts downregulates the activation of ER stress markers that occur due to viral protein overload; and (4) palmitate, a saturated fatty acid with similar carbon structure to palmitoleate augments cell death in ZIKV-infected trophoblasts. The schematic representation of palmitoleate protection against ZIKV-induced trophoblast apoptosis is shown in Figure 7.

ZIKV infection from the mother to the developing fetus is detrimental in causing congenital Zika syndrome [4]. Trophoblasts, the epithelial cells of the placenta express receptors, such as AXL, Tyro3, and T-cell immunoglobulin and mucin domain 1 (TIM1) that facilitate the entry of ZIKV into these cells [33,34]. ZIKV infection of *Infar1* knockout mice shows that ZIKV is able to breach the placental barrier and affects the survivability of the fetuses [12]. Similarly, a human STAT2 knock-in, immunocompetent mouse model shows that a mouse adapted ZIKV strain belonging to African lineage was able to cross the placental barrier and blood–brain barrier [35]. Therefore, transplacental route of transmission from mother to the fetus plays a crucial role in the disease process [36,37]. In our previous study, we showed that ZIKV induces a caspase-dependent trophoblast apoptosis as evidenced by significant increase in percent apoptotic nuclei and caspase 3/7 activation following ZIKV infection. Further, inhibition of caspases activity using Z-VAD-fmk prevented ZIKV-induced placental trophoblast apoptosis [30]. ZIKV is also known to cause changes in the sphingolipid metabolism in the host cells. Ceramide, a sphingolipid, is already known to be associated with apoptosis and has been found essential for ZIKV replication cycle in the host cells [38,39]. Ceramides are also known to cause ER stress and affect overall lipid metabolism in hepatocytes [40]. However, the protective role of palmitoleate supplementation against ZIKV-induced ER stress and apoptosis via alteration of sphingolipid metabolism needs further investigation.

There are several potential vaccine candidates, therapeutic drugs, and nutraceutical compounds under investigation for protection and treatment against ZIKV infection [41,42,43,44,45]. Since the prospective target population who needs protection during outbreaks involves pregnant women, this poses challenges regarding the safety of the vaccine candidates or the drugs that can ensure safety to both the mother and the developing baby without any adverse reactions [46,47,48,49,50]. In contrast, nutrient compounds can be an alternative strategy to combat viral infections in the context of safety during pregnancy. For example, 25-hydroxy cholesterol, an oxysterol metabolite, which plays a critical role in cholesterol biosynthesis and innate immune response, was shown to be protective against ZIKV-induced microcephaly in type I interferon α/β receptor knockout (*Infar*^−/−^) mice [51]. In an another study, it was observed that natural polyphenols like delphinidin and epigallocatechin gallate have anti-viral properties against flavivirus including ZIKV [52]. Curcumin, a polyphenol present in turmeric tubers, was also found to inhibit the attachment of ZIKV to the host cells [53]. Naringenin, a flavonoid compound seen in citrus fruits, had anti-viral properties against ZIKV infection by interacting with the protease domain of the virus [54]. In the present study, we established the protective role of palmitoleate against ZIKV-induced apoptosis in placental trophoblasts in an in vitro model.

Palmitoleate (16:1 n-7) is rich in dietary sources, such as sea buckthorn oil and macadamia nuts [55]. In mammals, palmitoleate can be synthesized by stearoyl-CoA desaturase 1 (SCD1) enzyme from the saturated fatty acid, palmitate [56]. Palmitoleate is abundant in adipose tissue, blood cells, and it is a part of cell membrane structure [57]. Palmitoleate plays an important role in maintaining metabolic health and homeostasis by acting as a lipokine [58]. Palmitoleate decreases fat deposition in the liver and enhances insulin sensitivity [57]. Studies have shown that palmitoleate protects against free fatty acid-induced hepatocyte lipoapoptosis [23] and trophoblast lipoapoptosis [59], respectively. Supplementation of palmitoleate has shown to be effective against non-alcoholic fatty liver disease and atherosclerosis in mouse models [60,61]. Palmitoleate can skew pro-inflammatory state to anti-inflammatory state of macrophages in mice fed with high fat diet via AMP activated protein kinase signaling [62]. Previous studies also suggest that monounsaturated fatty acids, such as palmitoleate and oleate, inhibit replication of enveloped bacteriophage phi6 and PR4 [63,64]. Further, palmitoleate was shown to protect against tunicamycin- and palmitate-induced ER stress and apoptosis in hepatocytes and pancreatic beta cells, respectively [23,24]. Our data in the present study support the protective role of palmitoleate against ZIKV-induced ER stress and apoptosis in placental trophoblasts and, therefore, could likely serve as a therapeutic candidate or preventive nutrient compound for ZIKV infection in pregnant mothers in disease infection prone areas.

Our data show proof that palmitoleate prevents ZIKV-induced trophoblast apoptosis. We also saw a reduction in the viral RNA copy number in the cell culture supernatant from JAR (0.1 and 1 MOI) and HTR-8 (1 MOI) infected cells following palmitoleate treatment. On the other hand, we saw a trend towards reduction viral RNA copy number in both JAR and HTR-8 cell lysate, this could be due to the fact that viral particles were already released from the cells to the culture supernatant. We also saw a reduction in the expression of viral E protein in JEG-3 cells infected with 0.1 MOI r-MRV and with a co-treatment of 200 µM palmitoleate. Similarly, 200 µM palmitoleate treatment showed improved cell survivability and reduced viral E protein staining, further suggests that it could interfere with the viral replication. Viral E protein in ZIKV also has a lipid component and might possibly be altered with palmitoleate treatment, which require further investigations.

Although we saw significant reduction in viral E gene copy number in 200 µM palmitate treated JEG-3 cell lysate, this might be due to the fact that there are less viable cells for viral replication in palmitate treated cells as observed in apoptotic nuclei and cell viability assay. The MTT assay measures active mitochondrial dehydrogenase and we showed that mitochondrial enzyme activity is compromised in ZIKV-infected cells alone, whereas palmitate treatment to ZIKV-infected cells resulted in intact mitochondrial enzyme activity. Further, to substantiate this phenomenon, a recent study suggested that low levels of palmitate supplementation can increase mitochondrial function [65]. Moreover, the use of crystal violet for cell viability assessment has been shown to be more reliable than MTT assay [66].

ZIKV is also known to alter the lipid homeostasis of the placenta by altering the organelles in the cells by forming virus replicating complexes enclosed in vesicles. ZIKV also resulted increase in the accumulation of large lipid droplets both in infected and uninfected bystander placental cells [15]. However further studies are required to elucidate the mechanism behind the protective role of palmitoleate by affecting the viral replication. This could be possible through ways such as (i) hindrance against Zika viral entry receptor binding; (ii) E protein lipid component; or (iii) inhibition of specialized viral replication complex.

Studies have shown that palmitate, a saturated fatty acid, can either promote viral infection [67] or have anti-viral properties [68] via autophagy flux mechanism. We found that palmitate treatment augmented apoptosis in ZIKV-infected trophoblasts. A study using influenza A virus model showed that palmitate supplementation to the cells can enhance viral replication [69]. Similarly, Rift Valley Fever virus-infected cells were shown to activate AMP-activated protein kinase (AMPK) and decrease viral replication by limiting fatty acid synthesis, treatment of palmitate helped in initiating fatty acid synthesis and aids in replication of the Rift Valley Fever virus [70]. In our study, palmitate treatment to ZIKV-infected cells reduces the cell viability, probably inducing an alternate cell death pathway in addition to apoptosis.

Palmitoleate is known to activate cell survival pathways and has been shown to protect against metabolic syndrome [57]. Palmitoleate plays a protective role by modulating peroxisome proliferator-activated receptor alpha (PPARα), an important transcription factor that regulates fatty acid oxidation by activating AMPK, which in turn improves glucose metabolism in liver cells of mice fed with high fat [56,71]. Further, post-translational modification of Wnt protein with palmitoylation results in a signaling pathway that activates β-catenin, a cell survival signal [72,73]. Several studies have also shown that palmitoleate supplementation helps in improving metabolic diseases in humans such as cardiovascular diseases and diabetes mellitus [74,75]. Our study results suggests that palmitoleate treatment in ZIKV-infected trophoblasts is protective against ER stress and apoptosis, thereby considerably improves cell survival. However further mechanistic studies are underway in elucidating the protective role of palmitoleate against ZIKV-induced trophoblast apoptosis.

## 5. Conclusions

Palmitoleate is protective against ZIKV-induced ER stress and apoptosis in trophoblasts. The mechanism of palmitoleate protection against ZIKV-induced ER stress and apoptosis is either via direct interference of viral replication or by the activation of cellular survival pathways, or a combination of both, which needs further investigations.

## Figures and Tables

**Table 1:** List of primers used.

Primer	Forward Primer	Reverse Primer	Product Length
*XBP1*	5′AAACAGAGTAGCAGC TCAGACTGC 3′	5′TCCTTCTGGGTAGAC CTCTGGGAG 3′	Unspliced forms ~474 bp Cleaved by the restriction endonuclease (PstI)—two products are around 296 bp and 183 bp are formed Spliced forms lack restriction enzyme site ~448 bp
*GAPDH*	5′AATCCCATCACCATC TTCCA 3′	5′TTCACACCCATGACG AAC AT 3′	~194 bp
*18srRNA*	5′CGTTCTTAGTTGGTG GAGCG 3′	5′CGCTGAGCCAGT CAG TGTAG 3′	~212 bp
*CHOP*	5′-ATGGCAGCTGAGTCATTGCCTTTC-3′	5′-AGAAGCAGGGTCAAGAGTGGTGAA-3	~265 bp
Viral Envelope	Forward 5′-GTCGTTGCCCAACACAAG-3′ Reverse 5′-CCACTAATGTTCTTTTGCAGAC-3′		
Hydrolysis probe used for viral E gene	5′-/56-FAM (5′ 6-carboxyfluorescein)/AGCCTACCT/ZEN/TGACAAGCAATCAGACACTCAA/3IABkFQ (3′ Iowa black fluorescent quencher)/-3′		

## Supplementary Materials

The following are available online at https://www.mdpi.com/article/10.3390/biomedicines9060643/s1, Supplementary Figure S1. (A) Increase in percentage apoptotic nuclei in JEG-3 cells infected with 1.0 MOI PRV 96 hpi when compared to uninfected vehicle cells. On treatment with 100–200 µM palmitoleate post-infection, there was significant reduction in the percent apoptotic nuclei. (B) Similarly, there was significant upregulation of caspase 3/7 in 1.0 MOI PRV infected JAR cells when compared to uninfected vehicle cells, but this was significantly downregulated when the cells were treated with 100–200 µM palmitoleate post-infection. Data represent mean ± SEM, *n* = 3. * *p* < 0.05 compared to uninfected vehicles cells, # *p* < 0.05 compared to ZIKV-infected cells. Supplementary Figure S2. (A) Plaque assay using cell culture supernatant showed a trend in decreased pfu/mL in 200 µM palmitoleate treated +ZIKV-infected cells. Similarly, 100 µM palmitate cells for 48 h with 0.1 MOI r-MRV showed a trend towards increase in pfu/mL but in contrast 200 µM palmitate treatment showed a trend towards reduction in pfu/mL. Data represent mean ± SEM, *n* = 3. # *p* < 0.05 compared to ZIKV-infected cells. (B) Similarly, viral envelope copy number in cell lysate with 200 µM palmitoleate treatment showed a trend towards reduction when compared 0.1 MOI r-MRV-infected cells alone but 200 µM palmitate treated cells showed a significant reduction in the viral envelope RNA copy number 48 hpi. Data represent mean ± SEM, *n* = 3. # *p* < 0.05 compared ZIKV-infected cells.

## References

[1] Dick G.W.A., Kitchen S.F., Haddow A.J. (1952). Zika Virus (I). Isolations and serological specificity. Trans. R. Soc. Trop. Med. Hyg..

[2] Weaver S.C., Costa F., Garcia-Blanco M.A., Ko A., Ribeiro G.S., Saade G., Shi P.-Y., Vasilakis N. (2016). Zika virus: History, emergence, biology, and prospects for control. Antivir. Res..

[3] Musso D., Gubler D.J. (2016). Zika Virus. Clin. Microbiol. Rev..

[4] Moore C.A., Staples J.E., Dobyns W.B., Pessoa A., Ventura C.V., Da Fonseca E.B., Ribeiro E.M., Ventura L.O., Neto N.N., Arena J.F. (2017). Characterizing the Pattern of Anomalies in Congenital Zika Syndrome for Pediatric Clinicians. JAMA Pediatr..

[5] Rosário M.S.D., De Siqueira I.C., Rodrigues S.G., Martins L.C., Vasilakis N., Novaes M.A.C., Alcantara L.C.J., Farias D.S., Jesus P.A., Ko A.I. (2016). Guillain–Barré Syndrome After Zika Virus Infection in Brazil. Am. J. Trop. Med. Hyg..

[6] Bhatnagar J., Rabeneck D.B., Martines R.B., Reagan-Steiner S., Ermias Y., Estetter L.B., Suzuki T., Ritter J., Keating M.K., Al J.B.E. (2017). Zika Virus RNA Replication and Persistence in Brain and Placental Tissue. Emerg. Infect. Dis..

[7] Quicke K.M., Bowen J.R., Johnson E.L., McDonald C.E., Ma H., O’Neal J.T., Rajakumar A., Wrammert J., Rimawi B.H., Pulendran B. (2016). Zika Virus Infects Human Placental Macrophages. Cell Host Microbe.

[8] Rabelo K., De Souza L.J., Salomão N.G., Machado L.N., Pereira P.G., Portari E.A., Basílio-De-Oliveira R., Dos Santos F.B., Neves L.D., Morgade L.F. (2020). Zika Induces Human Placental Damage and Inflammation. Front. Immunol..

[9] Simoni M.K., Jurado K.A., Abrahams V.M., Fikrig E., Guller S. (2017). Zika virus infection of Hofbauer cells. Am. J. Reprod. Immunol..

[10] Ribeiro M.R., Moreli J.B., Marques R.E., Papa M.P., Meuren L.M., Rahal P., De Arruda L.B., Oliani A.H., Oliani D.C.M.V., Oliani S.M. (2018). Zika-virus-infected human full-term placental explants display pro-inflammatory responses and undergo apoptosis. Arch. Virol..

[11] Sheridan M.A., Yunusov D., Balaraman V., Alexenko A.P., Yabe S., Verjovski-Almeida S., Schust D.J., Franz A.W., Sadovsky Y., Ezashi T. (2017). Vulnerability of primitive human placental trophoblast to Zika virus. Proc. Natl. Acad. Sci. USA.

[12] Miner J.J., Cao B., Govero J., Smith A.M., Fernandez E., Cabrera O.H., Garber C., Noll M., Klein R.S., Noguchi K.K. (2016). Zika Virus Infection during Pregnancy in Mice Causes Placental Damage and Fetal Demise. Cell.

[13] Seferovic M., Martín C.S.-S., Tardif S.D., Rutherford J., Castro E.C.C., Li T., Hodara V.L., Parodi L.M., Giavedoni L., Layne-Colon D. (2018). Experimental Zika Virus Infection in the Pregnant Common Marmoset Induces Spontaneous Fetal Loss and Neurodevelopmental Abnormalities. Sci. Rep..

[14] Brasil P., Pereira J.P., Moreira M.E., Nogueira R.M.R., Damasceno L., Wakimoto M., Rabello R.S., Valderramos S.G., Halai U.-A., Salles T.S. (2016). Zika Virus Infection in Pregnant Women in Rio de Janeiro. N. Engl. J. Med..

[15] Chen Q., Gouilly J., Ferrat Y.J., Espino A., Glaziou Q., Cartron G., El Costa H., Al-Daccak R., Jabrane-Ferrat N. (2020). Metabolic reprogramming by Zika virus provokes inflammation in human placenta. Nat. Commun..

[16] Shan C., Muruato A.E., Jagger B.W., Richner J., Nunes B.T.D., Medeiros D.B.A., Xie X., Nunes J.G.C., Morabito K.M., Kong W.-P. (2017). A single-dose live-attenuated vaccine prevents Zika virus pregnancy transmission and testis damage. Nat. Commun..

[17] Chahal J.S., Fang T., Woodham A.W., Khan O.F., Ling J., Anderson D.G., Ploegh H.L. (2017). An RNA nanoparticle vaccine against Zika virus elicits antibody and CD8+ T cell responses in a mouse model. Sci. Rep..

[18] La Rocca R.A., Abbink P., Peron J.P.S., Zanotto J.P.S.P.P.M.D.A., Iampietro M.J., Badamchi-Zadeh A., Boyd M., Ng’Ang’A D., Kirilova M., Nityanandam R. (2016). Vaccine protection against Zika virus from Brazil. Nature.

[19] Sumathy K., Kulkarni B., Gondu R.K., Ponnuru S.K., Bonguram N., Eligeti R., Gadiyaram S., Praturi U., Chougule B., Karunakaran L. (2017). Protective efficacy of Zika vaccine in AG129 mouse model. Sci. Rep..

[20] Xu M., Lee E.M., Wen Z., Cheng Y., Huang W.-K., Qian X., Tcw J., Kouznetsova J., Ogden S.C., Hammack C. (2016). Identification of small-molecule inhibitors of Zika virus infection and induced neural cell death via a drug repurposing screen. Nat. Med..

[21] Wilder-Smith A., Vannice K., Durbin A., Hombach J., Thomas S.J., Thevarjan I., Simmons C.P. (2018). Zika vaccines and therapeutics: Landscape analysis and challenges ahead. BMC Med..

[22] Sacramento C.Q., De Melo G.R., De Freitas C.S., Rocha N., Hoelz L.V.B., Miranda M., Fintelman-Rodrigues N., Marttorelli A., Ferreira A.C., Barbosa-Lima G. (2017). The clinically approved antiviral drug sofosbuvir inhibits Zika virus replication. Sci. Rep..

[23] Akazawa Y., Cazanave S., Mott J.L., Elmi N., Bronk S.F., Kohno S., Charlton M.R., Gores G.J. (2010). Palmitoleate attenuates palmitate-induced Bim and PUMA up-regulation and hepatocyte lipoapoptosis. J. Hepatol..

[24] Diakogiannaki E., Welters H.J., Morgan N.G. (2008). Differential regulation of the endoplasmic reticulum stress response in pancreatic β-cells exposed to long-chain saturated and monounsaturated fatty acids. J. Endocrinol..

[25] Lee D.M., Sevits K.J., Battson M.L., Wei Y., Cox-York K.A., Gentile C.L. (2019). Monounsaturated fatty acids protect against palmitate-induced lipoapoptosis in human umbilical vein endothelial cells. PLoS ONE.

[26] Annamalai A.S., Pattnaik A., Sahoo B.R., Muthukrishnan E., Natarajan S.K., Steffen D., Vu H.L.X., Delhon G., Osorio F.A., Petro T.M. (2017). Zika Virus Encoding Nonglycosylated Envelope Protein Is Attenuated and Defective in Neuroinvasion. J. Virol..

[27] Natarajan S.K., Ingham S.A., Mohr A.M., Wehrkamp C.J., Ray A., Roy S., Cazanave S.C., Phillippi M.A., Mott J.L. (2014). Saturated free fatty acids induce cholangiocyte lipoapoptosis. Hepatology.

[28] Cazanave S.C., Elmi N.A., Akazawa Y., Bronk S.F., Mott J.L., Gores G.J. (2010). CHOP and AP-1 cooperatively mediate PUMA expression during lipoapoptosis. Am. J. Physiol. Liver Physiol..

[29] Lu S., Natarajan S.K., Mott J.L., Kharbanda K.K., Harrison-Findik D.D. (2016). Ceramide Induces Human Hepcidin Gene Transcription through JAK/STAT3 Pathway. PLoS ONE.

[30] Muthuraj P.G., Sahoo P.K., Kraus M., Bruett T., Annamalai A.S., Pattnaik A., Pattnaik A.K., Byrareddy S.N., Natarajan S.K. (2021). Zika virus Infection Induces Endoplasmic Reticulum Stress and Apoptosis in Placental Trophoblasts. Cell Death Discov..

[31] Pattnaik A., Palermo N., Sahoo B.R., Yuan Z., Hu D., Annamalai A.S., Vu H.L., Correas I., Prathipati P.K., Destache C.J. (2018). Discovery of a non-nucleoside RNA polymerase inhibitor for blocking Zika virus replication through in silico screening. Antivir. Res..

[32] Feoktistova M., Geserick P., Leverkus M. (2016). Crystal Violet Assay for Determining Viability of Cultured Cells. Cold Spring Harb. Protoc..

[33] Tabata T., Petitt M., Puerta-Guardo H., Michlmayr D., Wang C., Fang-Hoover J., Harris E., Pereira L. (2016). Zika Virus Targets Different Primary Human Placental Cells, Suggesting Two Routes for Vertical Transmission. Cell Host Microbe.

[34] Aagaard K.M., Lahon A., Suter M.A., Arya R., Seferovic M.D., Vogt M.B., Hu M., Stossi F., Mancini M.A., Harris R.A. (2017). Primary Human Placental Trophoblasts are Permissive for Zika Virus (ZIKV) Replication. Sci. Rep..

[35] Gorman M.J., Caine E.A., Zaitsev K., Begley M.C., Weger-Lucarelli J., Uccellini M.B., Tripathi S., Morrison J., Yount B.L., Dinnon K.H. (2018). An Immunocompetent Mouse Model of Zika Virus Infection. Cell Host Microbe.

[36] Rombi F., Bayliss R., Tuplin A., Yeoh S. (2020). The journey of Zika to the developing brain. Mol. Biol. Rep..

[37] Mysorekar I.U. (2017). Zika Virus Takes a Transplacental Route to Infect Fetuses: Insights from an Animal Model. Mo. Med..

[38] Leier H.C., Weinstein J.B., Kyle J.E., Lee J.-Y., Bramer L.M., Stratton K.G., Kempthorne D., Navratil A.R., Tafesse E.G., Hornemann T. (2020). A global lipid map defines a network essential for Zika virus replication. Nat. Commun..

[39] Yager E.J., Konan K.V. (2019). Sphingolipids as Potential Therapeutic Targets against Enveloped Human RNA Viruses. Viruses.

[40] Kim Y.-R., Lee E.-J., Shin K.-O., Kim M.H., Pewzner-Jung Y., Lee Y.-M., Park J.-W., Futerman A.H., Park W.-J. (2019). Hepatic triglyceride accumulation via endoplasmic reticulum stress-induced SREBP-1 activation is regulated by ceramide synthases. Exp. Mol. Med..

[41] Diamond M.S., Ledgerwood J.E., Pierson T.C. (2019). Zika Virus Vaccine Development: Progress in the Face of New Challenges. Annu. Rev. Med..

[42] Pattnaik A., Sahoo B.R., Pattnaik A.K. (2020). Current Status of Zika Virus Vaccines: Successes and Challenges. Vaccines.

[43] Han Y., Mesplède T. (2018). Investigational drugs for the treatment of Zika virus infection: A preclinical and clinical update. Expert Opin. Investig. Drugs.

[44] Baz M., Boivin G. (2019). Antiviral Agents in Development for Zika Virus Infections. Pharmaceuticals.

[45] Wang L., Liang R., Gao Y., Li Y., Deng X., Xiang R., Zhang Y., Ying T., Jiang S., Yu F. (2019). Development of Small-Molecule Inhibitors Against Zika Virus Infection. Front. Microbiol..

[46] Poland G.A., Ovsyannikova I.G., Kennedy R.B. (2019). Zika Vaccine Development: Current Status. Mayo Clin. Proc..

[47] Barrett A.D.T. (2018). Current status of Zika vaccine development: Zika vaccines advance into clinical evaluation. npj Vaccines.

[48] Qureshi A.I. (2018). Chapter 10—Zika Virus Infection: Therapeutics. Zika Virus Disease.

[49] Bernatchez J.A., Tran L.T., Li J., Luan Y., Siqueira-Neto J.L., Li R. (2020). Drugs for the Treatment of Zika Virus Infection. J. Med. Chem..

[50] Weger-Lucarelli J., Auerswald H., Vignuzzi M., Dussart P., Karlsson E.A. (2018). Taking a bite out of nutrition and arbovirus infection. PLoS Negl. Trop. Dis..

[51] Li C., Deng Y.-Q., Wang S., Ma F., Aliyari R., Huang X.-Y., Zhang N.-N., Watanabe M., Dong H.-L., Liu P. (2017). 25-Hydroxycholesterol Protects Host against Zika Virus Infection and Its Associated Microcephaly in a Mouse Model. Immunity.

[52] Vázquez-Calvo Á., de Oya N.J., Martín-Acebes M.A., Garcia-Moruno E., Saiz J.-C. (2017). Antiviral Properties of the Natural Polyphenols Delphinidin and Epigallocatechin Gallate against the Flaviviruses West Nile Virus, Zika Virus, and Dengue Virus. Front. Microbiol..

[53] Mounce B.C., Cesaro T., Carrau L., Vallet T., Vignuzzi M. (2017). Curcumin inhibits Zika and chikungunya virus infection by inhibiting cell binding. Antivir. Res..

[54] Cataneo A.H.D., Kuczera D., Koishi A.C., Zanluca C., Silveira G.F., De Arruda T.B., Suzukawa A.A., Bortot L.O., Dias-Baruffi M., Verri W.A. (2019). The citrus flavonoid naringenin impairs the in vitro infection of human cells by Zika virus. Sci. Rep..

[55] Weimann E., Silva M.B.B., Murata G.M., Bortolon J.R., Dermargos A., Curi R., Hatanaka E. (2018). Topical anti-inflammatory activity of palmitoleic acid improves wound healing. PLoS ONE.

[56] De Souza C.O., Vannice G.K., Neto J.C.R., Calder P.C. (2018). Is Palmitoleic Acid a Plausible Nonpharmacological Strategy to Prevent or Control Chronic Metabolic and Inflammatory Disorders?. Mol. Nutr. Food Res..

[57] Frigolet M.E., Gutiérrez-Aguilar R. (2017). The Role of the Novel Lipokine Palmitoleic Acid in Health and Disease. Adv. Nutr..

[58] Hernández-Saavedra D., Stanford K.I. (2019). The Regulation of Lipokines by Environmental Factors. Nutrients.

[59] Natarajan S.K., Bruett T., Muthuraj P.G., Sahoo P.K., Power J., Mott J.L., Hanson C., Anderson-Berry A. (2021). Saturated free fatty acids induce placental trophoblast lipoapoptosis. PLoS ONE.

[60] Çimen I., Kocaturk B., Koyuncu S., Tufanli O., Onat U.I., Yıldırım A.D., Apaydın O., Demirsoy Ş., Aykut Z.G., Nguyen U.T. (2016). Prevention of atherosclerosis by bioactive palmitoleate through suppression of organelle stress and inflammasome activation. Sci. Transl. Med..

[61] Guo X., Li H., Xu H., Halim V., Zhang W., Wang H., Ong K.T., Woo S.-L., Walzem R.L., Mashek D.G. (2012). Palmitoleate Induces Hepatic Steatosis but Suppresses Liver Inflammatory Response in Mice. PLoS ONE.

[62] Chan K.L., Pillon N.J., Sivaloganathan D.M., Costford S.R., Liu Z., Théret M., Chazaud B., Klip A. (2015). Palmitoleate Reverses High Fat-induced Proinflammatory Macrophage Polarization via AMP-activated Protein Kinase (AMPK). J. Biol. Chem..

[63] Albert-Reinhardt S.C., Sands J.A. (1978). Inhibitory Effect of Fatty Acids on the Entry of the Lipid-Containing Bacteriophage PR4 into *Escherichia coli*. J. Virol..

[64] Sands J.A. (1977). Inactivation and Inhibition of Replication of the Enveloped Bacteriophage φ6 by Fatty Acids. Antimicrob. Agents Chemother..

[65] Liu L., Xie B., Fan M., Candas-Green D., Jiang J.X., Wei R., Wang Y., Chen H.-W., Hu Y., Li J.J. (2020). Low-Level Saturated Fatty Acid Palmitate Benefits Liver Cells by Boosting Mitochondrial Metabolism via CDK1-SIRT3-CPT2 Cascade. Dev. Cell.

[66] Śliwka L., Wiktorska K., Suchocki P., Milczarek M., Mielczarek S., Lubelska K., Cierpiał T., Łyżwa P., Kiełbasiński P., Jaromin A. (2016). The Comparison of MTT and CVS Assays for the Assessment of Anticancer Agent Interactions. PLoS ONE.

[67] Yu Y., Li C., Liu J., Zhu F., Wei S., Huang Y., Huang X., Qin Q. (2020). Palmitic Acid Promotes Virus Replication in Fish Cell by Modulating Autophagy Flux and TBK1-IRF3/7 Pathway. Front. Immunol..

[68] Librán-Pérez M., Pereiro P., Figueras A., Novoa B. (2019). Antiviral activity of palmitic acid via autophagic flux inhibition in zebrafish (Danio rerio). Fish. Shellfish. Immunol..

[69] Limsuwat N., Boonarkart C., Phakaratsakul S., Suptawiwat O., Auewarakul P. (2020). Influence of cellular lipid content on influenza A virus replication. Arch. Virol..

[70] Moser T.S., Schieffer D., Cherry S. (2012). AMP-Activated Kinase Restricts Rift Valley Fever Virus Infection by Inhibiting Fatty Acid Synthesis. PLoS Pathog..

[71] de Souza C.O., Teixeira A.A., Biondo L.A., Junior E.A.L., Batatinha H.A., Neto J.C.R. (2017). Palmitoleic Acid Improves Metabolic Functions in Fatty Liver by PPARα-Dependent AMPK Activation. J. Cell. Physiol..

[72] Rao D.M., Shackleford M.T., Bordeaux E.K., Sottnik J.L., Ferguson R.L., Yamamoto T.M., Wellberg E.A., Bitler B.G., Sikora M.J. (2019). Wnt family member 4 (WNT4) and WNT3A activate cell-autonomous Wnt signaling independent of porcupine O-acyltransferase or Wnt secretion. J. Biol. Chem..

[73] Takada R., Satomi Y., Kurata T., Ueno N., Norioka S., Kondoh H., Takao T., Takada S. (2006). Monounsaturated Fatty Acid Modification of Wnt Protein: Its Role in Wnt Secretion. Dev. Cell.

[74] Mozaffarian D., Cao H., King I.B., Lemaitre R.N., Song X., Siscovick D.S., Hotamisligil G.S. (2010). Trans-Palmitoleic Acid, Metabolic Risk Factors, and New-Onset Diabetes in U.S. Adults: A cohort study. Ann. Intern. Med..

[75] Stefan N., Kantartzis K., Celebi N., Staiger H., Machann J., Schick F., Cegan A., Elcnerova M., Schleicher E., Fritsche A. (2009). Circulating Palmitoleate Strongly and Independently Predicts Insulin Sensitivity in Humans. Diabetes Care.

