# Supplementary material for: Palmitoleate Protects against Zika Virus-Induced Placental Trophoblast Apoptosis
Source: Biomedicines. 2021 Jun 4;9(6):643. doi: 10.3390/biomedicines9060643 (PMC8226770; doi:10.3390/biomedicines9060643)
Supplement: Supplementary file 1 [file biomedicines-09-00643-s001.zip › biomedicines-1251084-supplementary.pdf]

Supplementary Material

Supplementary Figure 1

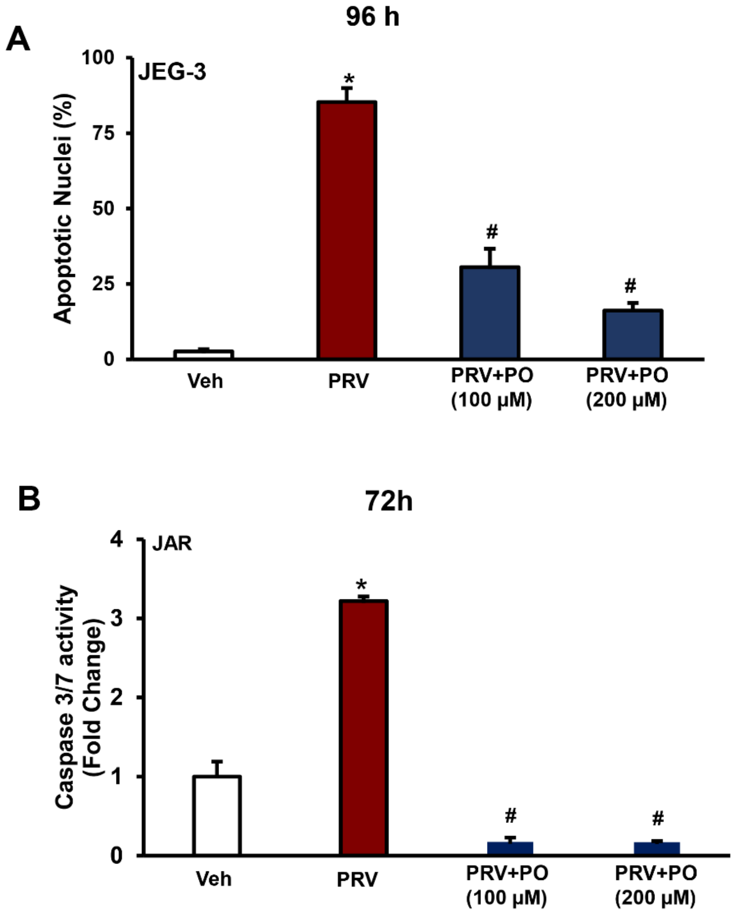

Figure S1. (**A**) Increase in percentage of apoptotic nuclei in JEG-3 cells infected with 1.0 MOI PRV 96hpi when compared to uninfected vehicle cells. On treatment with 100–200 µM palmitoleate post-infection, there was significant reduction in the percent apoptotic nuclei. (**B**) Similarly, there was significant upregulation of caspase 3/7 in 1.0MOI PRV infected JAR cells when compared to uninfected vehicle cells, but this was significantly downregulated when the cells were treated with 100–200 µM palmitoleate post-infection. Data represent mean ± SEM, $n = 3$. *$p < 0.05$ compared to uninfected vehicles cells, # $P < 0.05$ compared ZIKV infected cells.

Supplementary Figure-2

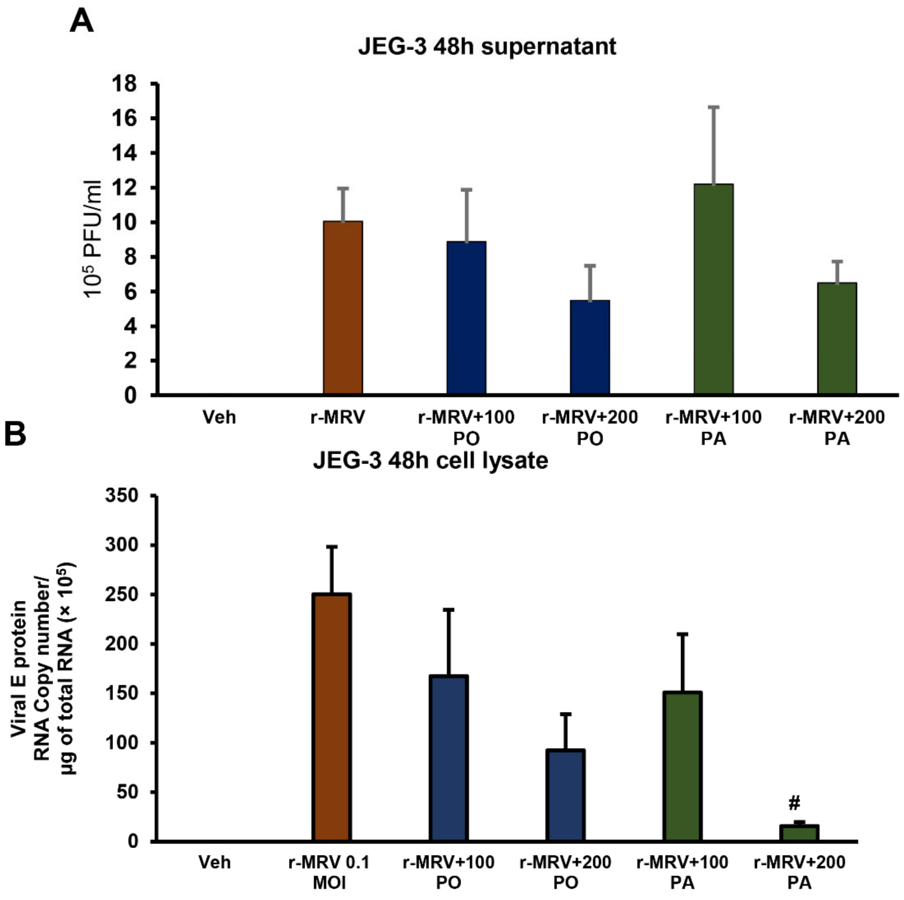

Figure S2. (**A**) Plaque assay using cell culture supernatant showed a trend in decreased pfu/ml in 200 µM palmitoleate treated +ZIKV infected cells. Similarly, 100 µM palmitate cells for 48 h with 0.1 MOI r-MRV showed a trend towards increase in pfu/ml, but, in contrast, 200 µM of palmitate treatment showed a trend towards reduction in pfu/ml. Data represent mean ± SEM, $n = 3$. #$p < 0.05$ compared ZIKV infected cells. (**B**) Similarly, the viral envelope copy number in cell lysate with 200 µM of palmitoleate treatment showed a trend towards reduction when compared to 0.1 MOI r-MRV infected cells alone, but 200 µM of palmitate-treated cells showed a significant reduction in the viral envelope RNA copy number 48 hpi. Data represent mean ±SEM, $n = 3$. #$p < 0.05$ compared to ZIKV infected cells.
